# Genetic scores of smoking behaviour in a Chinese population

**DOI:** 10.1038/srep22799

**Published:** 2016-03-07

**Authors:** Shanshan Yang, Yao He, Jianhua Wang, Yiyan Wang, Lei Wu, Jing Zeng, Miao Liu, Di Zhang, Bin Jiang, Xiaoying Li

**Affiliations:** 1 Institute of Geriatrics, Chinese PLA General Hospital, 28 Fuxing Road, Beijing, 100853, China; 2 Beijing Key Laboratory of Aging and Geriatrics, Chinese PLA General Hospital, 28 Fuxing Road, Beijing, 100853, China; 3 State Key Laboratory of Kidney Disease, Chinese PLA General Hospital, 28 Fuxing Road, Beijing, 100853, China; 4 Department of Chinese Traditional Medicine and Acupuncture, Chinese PLA General Hospital, 28 Fuxing Road, Beijing, 100853, China; 5 Department of Geriatric Cardiology, Chinese PLA General Hospital, 28 Fuxing Road, Beijing, 100853, China; 6 Jinan Military Area CDC, Jinan, Shandong, 250014, China

## Abstract

This study sought to structure a genetic score for smoking behaviour in a Chinese population. Single-nucleotide polymorphisms (SNPs) from genome-wide association studies (GWAS) were evaluated in a community-representative sample (N = 3,553) of Beijing, China. The candidate SNPs were tested in four genetic models (dominance model, recessive model, heterogeneous codominant model and additive model), and 7 SNPs were selected to structure a genetic score. A total of 3,553 participants (1,477 males and 2,076 females) completed the survey. Using the unweighted score, we found that participants with a high genetic score had a 34% higher risk of trying smoking and a 43% higher risk of SI at ≤18 years of age after adjusting for age, gender, education, occupation, ethnicity, body mass index (BMI) and sports activity time. The unweighted genetic scores were chosen to best extrapolate and understand these results. Importantly, genetic score was significantly associated with smoking behaviour (smoking status and SI at ≤18 years of age). These results have the potential to guide relevant health education for individuals with high genetic scores and promote the process of smoking control to improve the health of the population.

Smoke exposure is one of the most serious health problems worldwide^1^. Smoking creates a heavy disease burden and is associated with a 50% higher mortality rate from all causes among men who are smokers^2^. Active smoking is currently the most preventable cause of death, disability and various chronic diseases^3^,^4^,^5^,^6^,^7^,^8^,^9^. China is the largest tobacco grower and consumer in the world^10^, and the disease burden resulting from tobacco smoking is high^11^,^12^. One study recently conducted in East Asia demonstrated a smoking rate of 52.9% in adult Chinese men (aged 20–69 years) between 2008 and 2011^13^.

A study on twins in 2011 showed that susceptibility to smoking behaviour is influenced by genetic factors^14^, and family linkage analyses and candidate gene association studies have confirmed this finding^15^,^16^,^17^,^18^. Since 2005, genome-wide association studies (GWASs) of smoking behaviour (regular smoking, cigarettes per day and smoking initiation (SI) age) have identified 21 single-nucleotide polymorphisms (SNPs) with significant genome-wide associations (*P* < 5 × 10^−8^) in or near the following genes: *CHRNB3, CHRNA6, BDNF, CHRNA3, CHRNA5, AGPHD1, CHRNB4, CYP2A6* and *EGLN2*^19^,^20^,^21^,^22^,^23^,^24^,^25^,^26^. Many of these genes are expressed in or known to act in nicotine or dopamine receptor or brain-derived neurotrophic factor pathways.

Although GWASs have identified 21 SNPs associated with smoking behaviour^19^,^20^,^21^,^22^,^23^,^24^,^25^,^26^, each SNP accounted for only a very small fraction of the variation in smoking behaviour, and the results were unstable. The variable results among studies may be related to differences in effect sizes, sample sizes, genetic heterogeneity, genomic confounders, linkage disequilibrium (LD) and spurious associations^27^. Furthermore, the study populations of these GWASs did not include Chinese individuals. Thus, we conducted this study to verify these SNPs in a Chinese population and subsequently create a genetic score combining the effects of these SNPs on smoking behaviour.

## Design and Methods

### Study sample

**Figure 1:**
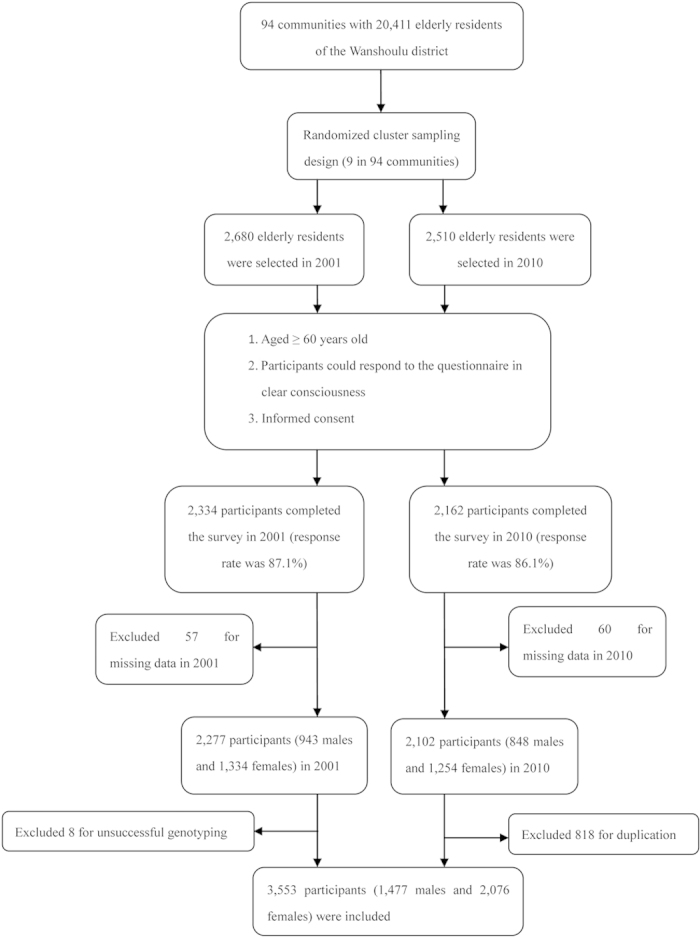
Flow diagram of the study population.

We conducted two population-based, cross-sectional surveys in 2001 and 2010 on elderly residents (aged ≥60 years) of the Wanshoulu district. As described in our previous study^28^,^29^, a 2-step randomized cluster sampling method was used to select 2,277 participants (943 males and 1,334 females) in 2001 and 2,102 participants (848 males and 1,254 females) in 2010. After excluding 818 participants duplicated in both surveys and 8 unsuccessful genotyping results, a total of 3,553 participants (1,477 males and 2,076 females) were included as our study sample (Fig. 1). Trained interviewers met with the participants face-to-face to complete a standardized questionnaire addressing a range of demographic factors, medical history and health-related behaviours (particularly smoking exposure status).

### Measurement of smoking behaviour

A smoker was defined as a person who had ever smoked a tobacco product daily for at least 6 months^30^. A heavy smoker was defined as a person who had ever smoked more than 20 cigarettes per day^31^. Additionally, an SI age of ≤18 years was used a measurement of smoking behaviour^1^ because previous studies have shown that compared with SI during adulthood, tobacco use prior to 18 years of age leads to behavioural consequences (such as drug abuse) during adulthood, in addition to more serious health consequences (including mental and physical effects)^32^.

### Measurement of covariates

The categories of educational attainment included 0–6 years (primary school or less), 6–12 years (middle school to high school or the equivalent) and ≥13 years (completed a university or other tertiary education). The occupation types were classified into the following three categories: white collar (professional, government), light physical labour (skilled worker, service, merchant) and hard physical labour (farmer, factory worker, manufacturing and transportation worker). Ethnicity was classified into the following two categories: Han and minority. Body mass index (BMI) was classified into the following three categories: normal (<24.00), overweight (24.00–27.99) and obese (≥ 28.00)^33^. Sports activity time was classified into the following three categories: <1 hour/week, 1–4 hours/week, and >4 hours/week.

### Genotyping

The standard proteinase K-phenol-chloroform method was used to extract DNA from whole peripheral blood samples. The laboratory staff was blinded to the identities of the subjects and their smoking status.

**Figure 2:**
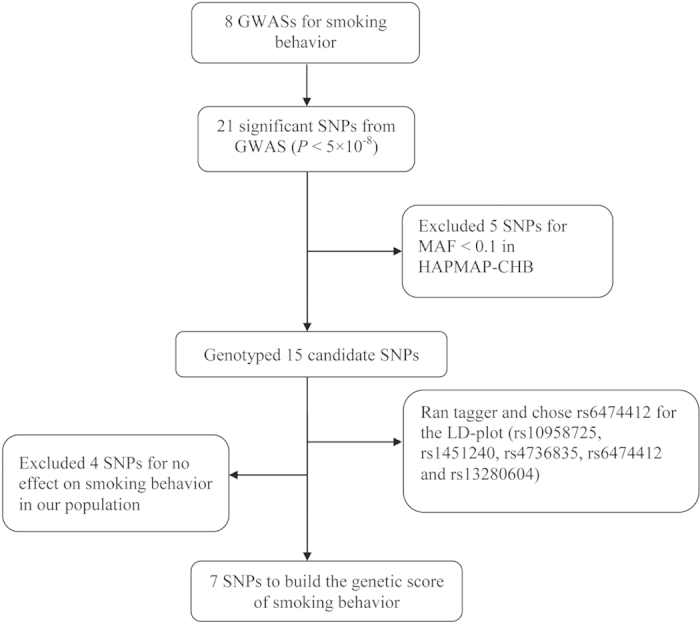
The process for choosing candidate SNPs.

Among the 21 previously reported SNPs, we excluded rs1051730, rs879048, rs2036527, rs8034191, rs11638372 and rs16969968 due to minor allele frequencies (MAFs) <0.1 in the HAPMAP-CHB (Chinese Han Beijing) population (Supplementary Table S1); however, the 15 remaining candidate SNPs were included in our analysis (Fig. 2). The MassARRAY system was used to genotype the candidate SNPs.

### Genetic score

Genotyping revealed an LD plot (Supplementary Fig. S1) for the 15 SNPs: using run tagger, we chose rs6474412 to represent this LD plot (Supplementary Table S2). To evaluate the effects of these SNPs on smoking behaviour, we examined the SNPs in four genetic models (dominance model, recessive model, heterogeneous codominant model and additive model^34^) and in males and females separately. We then excluded the SNPs with no significant effect on smoking behaviour in our population. The final genetic score was built on 7 SNPs (Supplementary Tables S3–9).

Similar to previous studies that evaluated genetic scores for smoking behaviour^35^ and obesity^36^, our genetic score was based on 3 methods. In the first two methods, each SNP was weighted according to the size of its relative effect (β coefficient) using two types of β coefficints: β1 was derived from our population and adjusted for demographic characteristics (age, gender, education, occupation and ethnicity), BMI and sports activity time; β2 was derived from the results of GWASs and meta-analyses (Table 1)^19^,^20^,^21^,^22^,^23^,^24^,^25^,^26^. The third method used the unweighted counts of risk alleles to construct the score.

### Statistical analysis

HAPLOVIEW software version 4.2 (http:// www.broadinstitute.org/haploview) was used for analyses of Hardy-Weinberg equilibrium (HWE), LD and run tagger. SPSS version 19.0 (serial No. 5076595) was used for the data analysis. The significance level for all tests was set at a two-tailed α value of 0.05. The differences in means and proportions were tested using t-tests and chi-squared tests, respectively. Logistic regression models were used to identify the odds ratio (OR) of the genetic score for smoking behaviour.

### Ethical considerations

The committee for medical ethics of the Chinese PLA General Hospital examined and approved our study; this study was performed in accordance with the ethical guidelines of the Declaration of Helsinki (version 2002). Each study participant provided written informed consent prior to completing the questionnaire.

## Results

### Patient characteristics

A total of 3,553 participants (1,477 males and 2,076 females) were included in our study. The average age was 70.29 ± 6.43 years. There were 1,067 smokers and 2,486 never smokers in our sample population: the two groups differed in gender (*P* < 0.001) and education (*P* = 0.007) but no significant differences were detected in age, ethnicity, occupation, BMI and sports activity time (*P* > 0.05) (Table 2). Table 3 depicts the genotype frequencies of the 7 SNPs.

### Effect of genetic score on smoking behaviour

#### Genetic score type 1

Risk alleles from the imputed data (0, 1 or 2) for each SNP were weighted according to their relative β coefficients (β1, Table 1), which were estimated from our data after adjusting for demographic characteristics (age, gender, education, occupation and ethnicity), BMI and sports activity time. Weighted risk alleles were summed for each individual to generate a type 1 genetic score representing the individual’s risk allele score (ranging from 0.06 to 0.88; average: 0.42 ± 0.14). The participants were divided into three groups according to tertiles (0.36 and 0.48): group 1 included participants with a genetic score <0.36; group 2 comprised participants with a genetic score 0.36–0.48; and group 3 included participants with a genetic score >0.48.

Through logistic regression analysis, we found that participants with a high genetic score (group 3) had a 26% higher risk of trying smoking and a 29% higher risk for SI at ≤18 years old after adjusting for age, gender, education, occupation, ethnicity, BMI and sports activity time. Among males, the ORs were even higher (1.37 and 1.37, respectively), whereas in females, the association was not significant (Table 4).

#### Genetic score type 2

Risk alleles from the imputed data (0, 1 or 2) per SNP were weighted for their relative β coefficients (β2, Table 1), which were estimated from previously reported GWASs and meta-analyses. Weighted risk alleles were summed for each individual to generate the type 2 genetic score representing the individual’s risk allele score (ranging from 0.14 to 3.53; average: 1.54 ± 0.70). The participants were divided into three groups according to tertiles (1.05 and 1.95): group 1 had a genetic score <1.05; group 2 had a genetic score of 1.05–1.95; and group 3 had a genetic score >1.95.

Regarding the type 2 genetic score, we found that participants with a high genetic score (group 3) had a 24% higher risk of trying smoking and a 28% higher risk for SI at ≤18 years of age after adjusting for age, gender, education, occupation, ethnicity, BMI and sports activity time. Among males, the ORs were even higher (1.37 and 1.42, respectively), whereas in females, the association was not significant (Table 5).

#### Genetic score type 3

Risk alleles from the imputed data (0, 1 or 2) per SNP were unweighted and summed for each individual, generating the type 3 genetic score as a representation of the individual’s risk allele score (ranging from 2 to 14; average: 7.47 ± 1.80). The participants were divided into three groups according to tertiles (7 and 9): group 1 had a genetic score <7; group 2 had a genetic score of 7–9; and group 3 had a genetic score >9.

Regarding the type 3 genetic score, we found that participants with a high genetic score (group 3) had a 34% higher risk of trying smoking and a 43% higher risk for SI at ≤18 years of age after adjusting for age, gender, education, occupation, ethnicity, BMI and sports activity time. Among males, the ORs were even higher (1.42 and 1.46, respectively), whereas in females, the association was not significant (Table 6).

### Receiver-operating characteristic (ROC) curves

**Figure 3:**
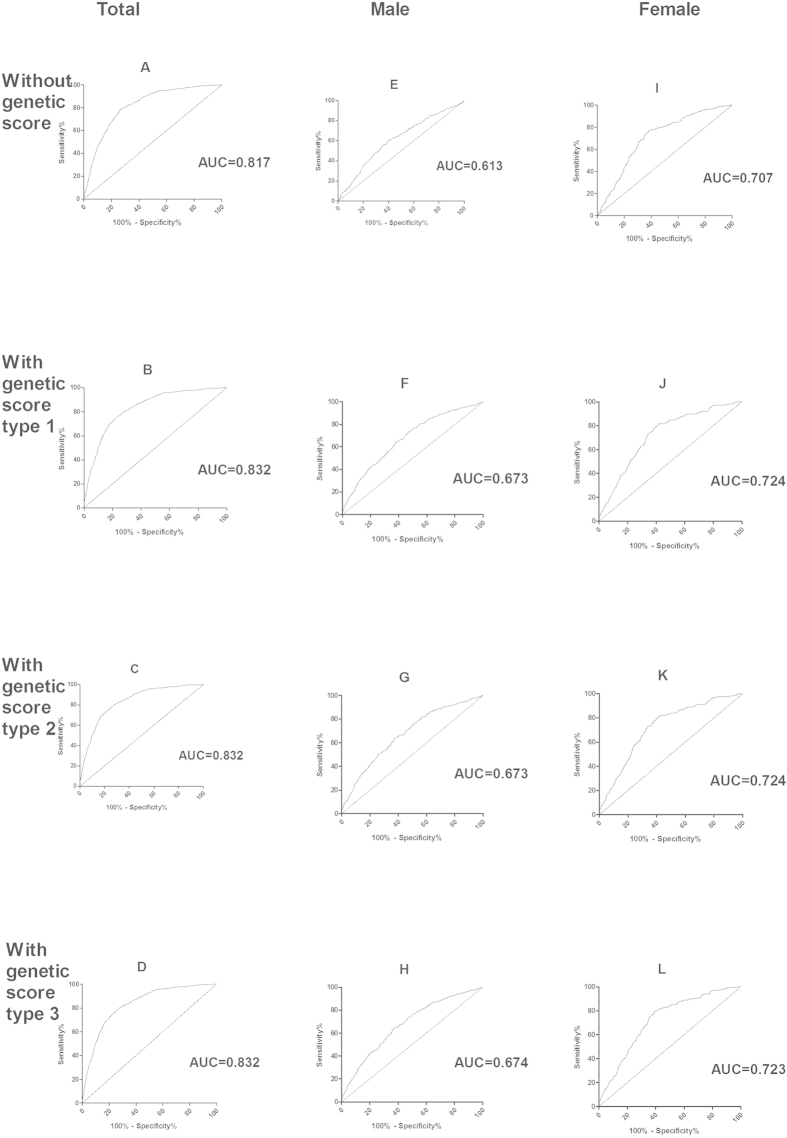
ROC curves of the four prediction models in total, male and female populations.

ROC curves were constructed using age, gender, education, occupation, ethnicity, BMI and sports activity time in addition to genetic score types 1, 2 and 3 (Fig. 3). The areas under the curve (AUCs) of the three types of genetic scores were 0.832, 0.832 and 0.832 for predicting smoking status in the total population; 0.673, 0.673 and 0.674 in males; and 0.724, 0.724 and 0.723 in females, respectively (Fig. 3). These results indicated that the associations of the three types of genetic scores with smoking were similar. Furthermore, for better extrapolation and improved understanding of such results, the unweighted genetic score represents the ideal choice.

Next, we compared the AUCs of age, gender, education, occupation, ethnicity, BMI and sports activity time with and without the genetic score (unweighted). These values were 0.832 and 0.817 in the total population, 0.674 and 0.613 in males, and 0.723 and 0.707 in females, respectively. This difference was significant in males (*P* < 0.05) (Fig. 3).

Furthermore, the average scores of the smoking group, heavy smoking group and SI at ≤18 years of age group were significantly higher than the never smoking group of males and the total population (Table 7).

## Discussion

In this study, we retested all 18 significant SNPs (*P* < 5 × 10^−8^) from GWASs conducted on smoking behaviour (cigarettes smoked per day (CPD), SI) in a Chinese population; we then chose 7 of these SNPs to derive genetic scores. We derived three types of genetic scores to evaluate the genetic risk of smoking behaviour (smoking, heavy smoking and SI at ≤18 years of age) and found that the evaluation capacities of these three scores were approximately the same. Furthermore, we linked genetic risk and smoking behaviour (smoking, heavy smoking and SI at ≤18 years of age) in a Chinese population.

Certain SNPs were significant in GWASs conducted in European or African American populations; however, the MAFs of these SNPs in the Chinese population were too low for our analysis. Furthermore, of the 15 candidate SNPs, 4 SNPs displayed no association with smoking behaviour in the Chinese population, although significant associations were found in GWASs conducted on other populations. We identified 7 SNPs that impacted the susceptibility to smoking behaviour in the Chinese population (similar to the reported GWAS results). Moreover, both our study and previous studies found SNPs with common and unique features in terms of MAF, haplotype blocks and effects in different populations^37^,^38^.

Previous genetic score studies have used two methods to create the genetic score: 1) summing the unweighted SNPs^35^ and 2) summing SNPs weighted by their effect^36^. To our knowledge, this study is the first to compare the effects of different genetic score generation methods, and we found that the three types of genetic scores elicited similar effects on smoking behaviour.

Furthermore, we found that genetic score was significantly associated with smoking behaviour (smoking status or SI at ≤ 18 years of age) in the Chinese population. This result is consistent with that of a study performed in New Zealand^35^, in which individuals with elevated genetic risk were more likely to convert to daily smoking as teenagers and progressed more rapidly from SI to heavy smoking.

However, the present study has several limitations. First, the candidate SNPs that were chosen from the GWAS results were mainly identified in European or African American populations; only a few such studies have been reported in Chinese populations. This may have decreased the reliability of the findings regarding SNPs related to smoking behaviour in the Chinese population. In addition, the SNPs from the USA/Northern European populations may not be suitable or sufficient to create a genetic score in the Chinese population. Thus, additional GWAS studies of large samples from the Chinese population should be conducted to create a more suitable genetic score for this population. Second, the small sample size of smoking women in our study may have decreased the stability of the results in women. Third, the genetic score created in our study requires verification in a larger Chinese sample.

To conclude, in this study, we tested GWAS-significant SNPs associated with smoking behaviour in a Chinese population and structured three types of genetic scores. We found that the effects of the three types of genetic score were similar; however, to best extrapolate and understand these types of results, the unweighted genetic score represents the ideal choice. Furthermore, the genetic score was significantly associated with smoking behaviour (smoking status and SI at ≤18 years of age). The results of this study may guide relevant health education for those with a high genetic score and promote smoking control to improve the health of the population.

## Additional Information

**How to cite this article**: Yang, S. *et al.* Genetic scores of smoking behaviour in a Chinese population. *Sci. Rep.* **6**, 22799; doi: 10.1038/srep22799 (2016).

## Supplementary Material

Supplementary Information

## Figures and Tables

**Table 1:** The 7 SNPs used to calculate the genetic score for smoking behaviour.

Gene	SNP	Alleles	Chromosome	Chromosomal location	β_1_*	β_2_#
CHRNB3	rs6474412	C/T	8	42669655	0.031	0.290^19^
BNDF	rs4923457	A/T	11	27605156	0.014	0.011^20^
LOC100188947	rs1329650	T/G	10	93338100	0.087	0.059^20^
CHRNA3	rs6495308	C/T	15	76694711	0.026	0.073^21^
CHRNA3	rs12914385	C/T	15	76685778	0.117	1.024^20^
HYKK	rs2036534	C/T	15	76614003	0.071	0.069^22^
EGLN2	rs7937	C/T	19	45994546	0.095	0.240^19^

^*^β_1_ was derived from our population and adjusted by demographic characteristics (age, gender, education, occupation and ethnicity), BMI and sports activity time.

^#^β_2_ was derived from GWASs and meta-analyses.

**Table 2:** Baseline characteristics of the participants.

	Total	Smokers	Never smokers
	(n = 3,553)	(n = 1,067)	(n = 2,486)
Age (years)			
<70	1,627 (45.8)	469 (44.0)	1,158 (46.6)
70–79	1,641 (46.2)	504 (47.2)	1,137 (45.7)
≥80	285 (8.0)	94 (8.8)	191 (7.7)
Gender			
Male	1,477 (41.6)	832 (78.0)	645 (25.9)
Female	2,076 (58.4)	235 (22.0)	1841 (74.1)
Ethnic group			
Han	3,479 (97.9)	1,049 (98.3)	2,430 (97.8)
Minority	74 (2.1)	18 (1.7)	56 (2.2)
Education level (years)			
0–6	1,225 (34.5)	405 (38.0)	820 (33.0)
7–12	1,333 (37.5)	365 (34.2)	968 (38.9)
13+	995 (28.0)	297 (27.8)	698 (28.1)
Occupation			
White collar	1,409 (39.7)	409 (38.3)	1,000 (40.2)
Light physical labour	1,402 (39.5)	420 (39.4)	982 (39.5)
Hard physical labour	742 (20.9)	238 (22.3)	504 (20.3)
BMI			
<24.00	1,280 (36.0)	370 (34.7)	910 (36.6)
24.00–27.99	1,585 (44.6)	483 (45.3)	1,102 (44.3)
≥28.00	688 (19.4)	214 (20.1)	474 (19.1)
Sports activity time			
<1 hour/week	710 (20.0)	222 (20.8)	488 (19.6)
1–4 hours/week	2151 (60.5)	628 (58.9)	1,523 (61.3)
>4 hours/week	692 (19.5)	217 (20.3)	475 (19.1)

**Table 3:** Genotype frequencies of the 7 SNPs.

	Total	Smoker	Never smoker
	(n = 3,553)	(n = 1,067)	(n = 2,486)
rs12914385			
CC	1,935 (54.5)	557 (52.2)	1,378 (55.4)
CT	1,391 (39.2)	431 (40.4)	960 (38.6)
TT	227 (6.4)	79 (7.4)	148 (6.0)
rs2036534			
CC	627 (17.6)	173 (16.2)	454 (18.3)
CT	1,749 (49.2)	540 (50.6)	1,209 (48.6)
TT	1,177 (33.1)	354 (33.2)	823 (33.1)
rs7937			
CC	375 (10.6)	99 (9.3)	276 (11.1)
CT	1,538 (43.3)	467 (43.8)	1,071 (43.1)
TT	1640 (46.2)	501 (47.0)	1,139 (45.8)
rs6474412			
CC	162 (4.6)	45 (4.2)	117 (4.7)
CT	1,163 (32.7)	356 (33.4)	807 (32.5)
TT	2,228 (62.7)	666 (62.4)	1,562 (62.8)
rs6495308			
TT	317 (8.9)	83 (7.8)	234 (9.4)
CT	1,456 (41.0)	445 (41.7)	1,011 (40.7)
CC	1,780 (50.1)	539 (50.5)	1,241 (49.9)
rs1329650			
AA	1,817 (51.1)	532 (49.9)	1,285 (51.7)
CA	1,401 (3.4)	430 (40.3)	971 (39.1)
CC	335 (9.4)	105 (9.8)	230 (9.3)
rs4923457			
TT	637 (17.9)	191 (17.9)	446 (17.9)
AT	1,793 (50.5)	538 (50.4)	1,255 (50.5)
AA	1,123 (31.6)	338 (31.7)	785 (31.6)

**Table 4:** Effect of genetic score 1 (β_1_) on smoking behaviour.

	Total			Male			Female		
	Smoker	Heavy smoker	SI ≤18	Smoker	Heavy smoker	SI ≤18	Smoker	Heavy smoker	SI ≤18
Group 1^a^	1	1	1	1	1	1	1	1	1
Group 2^a^	1.09 (0.91–1.30)	1.13 (0.87–1.45)	1.08 (0.89–1.32)	1.16 (0.90–1.49)	1.02 (0.76–1.37)	1.11 (0.86–1.44)	0.87 (0.62–1.21)	1.57(0.72–3.41)	0.87(0.58–1.31)
Group 3^a^	**1.23 (1.03**–**1.46)**	1.21 (0.94–1.56)	**1.28 (1.05**–**1.55)**	**1.35 (1.04**–**1.73)**	1.12 (0.84–1.50)	**1.37 (1.06**–**1.77)**	1.06 (0.77–1.46)	1.68 (0.78–3.62)	1.08 (0.73–1.60)
*P* for trend	**0.023**	0.135	**0.013**	**0.022**	0.437	**0.015**	0.743	0.189	0.693
Group 1^b^	1	1	1	1	1	1	1	1	1
Group 2^b^	1.08 (0.88–1.33)	1.14 (0.86–1.50)	1.06 (0.85–1.32)	1.23 (0.95–1.59)	1.08(0.80–1.45)	1.14 (0.88–1.47)	0.87 (0.61–1.23)	1.59 (0.73–3.46)	0.87(0.58–1.32)
Group 3^b^	**1.26 (1.03**–**1.55)**	1.20 (0.91–1.58)	**1.29 (1.04**–**1.60)**	**1.38 (1.06**–**1.78)**	1.13 (0.84–1.52)	**1.37 (1.06**–**1.77)**	1.08 (0.78–1.52)	1.75(0.81–3.78)	1.11 (0.74–1.64)
*P* for trend	**0.026**	0.192	**0.019**	**0.016**	0.408	**0.015**	0.654	0.161	0.63
Group 1^c^	1	1	1	1	1	1	1	1	1
Group 2^c^	1.08 (0.88–1.33)	1.14 (0.86–1.50)	1.06 (0.85–1.32)	1.23 (0.95–1.59)	1.08(0.80–1.46)	1.14 (0.88–1.47)	0.86(0.61–1.22)	1.59 (0.73–3.46)	0.87 (0.58–1.32)
Group 3^c^	**1.26 (1.03**–**1.56)**	1.19 (0.91–1.57)	**1.29 (1.04**–**1.60)**	**1.37 (1.06**–**1.78)**	1.12(0.84–1.51)	**1.37 (1.06**–**1.76)**	1.09 (0.78–1.52)	1.76(0.81–3.82)	1.11 (0.75–1.65)
*P* for trend	**0.026**	0.207	**0.020**	**0.016**	0.444	**0.017**	0.646	0.154	0.615

^a^Unadjusted.

^b^Adjusted for demographic characteristics (age, gender, education, occupation and ethnicity).

^c^Adjusted for demographic characteristics (age, gender, education, occupation and ethnicity), BMI and sports activity time.

SI = age of smoking initiation.

**Table 5:** Effect of genetic score 2 (β_2_) on smoking behaviour.

	Total			Male			Female		
	Smoker	Heavy smoker	SI ≤18	Smoker	Heavy smoker	SI ≤18	Smoker	Heavy smoker	SI ≤18
Group 1^a^	1	1	1	1	1	1	1	1	1
Group 2^a^	1.01 (0.85–1.21)	0.98 (0.76–1.27)	1.08 (0.89–1.32)	1.02 (0.79–1.31)	0.90 (0.67–1.21)	1.11 (0.86–1.43)	0.86 (0.61–1.20)	1.16 (0.61–2.65)	0.90 (0.61–1.35)
Group 3^a^	**1.22 (1.03**–**1.45)**	1.26 (0.98–1.61)	**1.28 (1.05**–**1.55)**	**1.32 (1.03**–**1.71)**	1.12 (0.84–1.49)	**1.41 (1.10**–**1.82)**	1.04 (0.75–1.44)	2.05 (0.51–4.29)	0.96 (0.65–1.43)
*P* for trend	**0.024**	0.066	**0.013**	**0.029**	0.427	**0.007**	0.831	**0.046**	0.838
Group 1^b^	1	1	1	1	1	1	1	1	1
Group 2^b^	1.00 (0.81–1.23)	0.98 (0.74–1.31)	1.07 (0.86–1.33)	1.09 (0.84–1.41)	0.96 (0.71–1.29)	1.14 (0.88–1.48)	0.84 (0.59–1.19)	1.16 (0.51–2.67)	0.90 (0.60–1.34)
Group 3^b^	**1.24 (1.01**–**1.52)**	1.24 (0.95–1.63)	**1.28(1.03**–**1.58)**	**1.37 (1.06**–**1.77)**	1.14 (0.85–1.52)	**1.42 (1.10**–**1.84)**	1.03 (0.74–1.44)	**2.10 (1.00**–**4.42)**	0.95 (0.64–1.43)
*P* for trend	**0.038**	0.108	**0.025**	**0.018**	0.382	**0.006**	0.863	**0.042**	0.808
Group 1^c^	1	1	1	1	1	1	1	1	1
Group 2^c^	1.00 (0.81–1.23)	0.99 (0.74–1.31)	1.07 (0.86–1.33)	1.09 (0.84–1.41)	0.96 (0.71–1.30)	1.14 (0.88–1.48)	0.84 (0.59–1.19)	1.16 (0.51–2.67)	0.90 (0.60–1.35)
Group 3^c^	**1.24 (1.01**–**1.52)**	1.24 (0.94–1.62)	**1.28 (1.03**–**1.58)**	**1.37 (1.06**–**1.77)**	1.13 (0.84–1.51)	**1.42 (1.10**–**1.83)**	1.03 (0.74–1.44)	**2.11 (1.00**–**4.44)**	0.96 (0.64–1.43)
*P* for trend	**0.039**	0.119	**0.026**	**0.018**	0.413	**0.007**	0.876	**0.041**	0.815

^a^Unadjusted.

^b^Adjusted for demographic characteristics (age, gender, education, occupation and ethnicity).

^c^Adjusted for demographic characteristics (age, gender, education, occupation and ethnicity), BMI and sports activity time.

SI = age of smoking initiation.

**Table 6:** Effect of genetic score 3 (unweighted) on smoking behaviour.

	Total			Male			Female		
	Smoker	Heavy smoker	SI ≤18	Smoker	Heavy smoker	SI ≤18	Smoker	Heavy smoker	SI ≤18
Group 1^a^	1	1	1	1	1	1	1	1	1
Group 2^a^	1.05 (0.88–1.24)	0.98 (0.77–1.26)	1.05 (0.86–1.27)	1.15 (0.90–1.47)	0.98 (0.74–1.31)	1.10 (0.86–1.42)	1.08 (0.78–1.51)	1.59 (0.72–3.51)	1.15 (0.77–1.73)
Group 3^a^	**1.29 (1.07**–**1.55)**	1.17 (0.90–1.52)	**1.40 (1.14**–**1.72)**	**1.39 (1.07**–**1.81)**	1.06 (0.78–1.43)	**1.47 (1.12**–**1.91)**	1.19 (0.83–1.70)	1.88 (0.82–4.33)	1.33 (0.86–2.06)
*P* for trend	**0.008**	0.253	**0.001**	**0.015**	0.714	**0.005**	0.348	0.141	0.194
Group 1^b^	1	1	1	1	1	1	1	1	1
Group 2^b^	1.10 (0.90–1.35)	1.01 (0.77–1.33)	1.10 (0.89–1.36)	1.14 (0.89–1.47)	0.96 (0.72–1.28)	1.10 (0.85–1.41)	1.04 (0.74–1.46)	1.52 (0.68–3.37)	1.12 (0.74–1.68)
Group 3^b^	**1.34 (1.08**–**1.67)**	1.15 (0.86–1.53)	**1.44 (1.14**–**1.81)**	**1.42 (1.09**–**1.86)**	1.07 (0.79–1.45)	**1.47 (1.13**–**1.92)**	1.19 (0.82–1.72)	1.88 (0.81–4.36)	1.34 (0.86–2.07)
*P* for trend	**0.008**	0.346	**0.002**	**0.011**	0.682	**0.005**	0.360	0.141	0.198
Group 1^c^	1	1	1	1	1	1	1	1	1
Group 2^c^	1.10 (0.90–1.35)	1.01 (0.77–1.33)	1.10 (0.88–1.36)	1.14 (0.89–1.47)	0.96 (0.72–1.28)	1.10 (0.85–1.41)	1.04 (0.74–1.46)	1.51 (0.68–3.37)	1.12 (0.74–1.68)
Group 3^c^	**1.34 (1.08**–**1.67)**	1.14 (0.85–1.52)	**1.43 (1.14**–**1.80)**	**1.42 (1.08**–**1.86)**	1.06 (0.78–1.43)	**1.46 (1.12**–**1.91)**	1.19 (0.82–1.72)	1.89 (0.81–4.37)	1.34 (0.86–2.08)
*P* for trend	**0.008**	0.375	**0.002**	**0.011**	0.735	**0.005**	0.369	0.140	0.194

^a^Unadjusted.

^b^Adjusted for demographic characteristics (age, gender, education, occupation and ethnicity).

^c^Adjusted for demographic characteristics (age, gender, education, occupation and ethnicity), BMI and sports activity time.

SI = age of smoking initiation.

**Table 7:** Average scores of never smokers, smokers, heavy smokers and SI at ≤18 years group.

	Never smoking	Smoking		Heavy smoking		SI ≤18 years	
	Mean ± SD	Mean ± SD	*P*	Mean ± SD	*P*	Mean ± SD	*P*
Total	7.43 ± 1.81	7.27 ± 1.78	0.029	7.55 ± 1.85	0.196	7.64 ± 1.80	0.006
Male	7.38 ± 1.82	7.58 ± 1.81	0.035	7.49 ± 1.86	0.346	7.64 ± 1.83	0.012
Female	7.44 ± 1.80	7.52 ± 1.64	0.511	8.02 ± 1.68	0.034	7.59 ± 1.67	0.312

*P* values were determined in comparison with the never smoked group.
