# Supplementary material for: Genetic scores of smoking behaviour in a Chinese population
Source: Sci Rep. 2016 Mar 7;6:22799. doi: 10.1038/srep22799 (PMC4780027; doi:10.1038/srep22799)
Supplement: Supplementary Information [file srep22799-s1.pdf]

Genetic scores of smoking behaviour in a Chinese population

Shanshan Yang [1,2,6], Yao He [1,2,3,*], Jianhua Wang [1,2], Yiyan Wang [1,2], Lei Wu [1,2], Jing Zeng [1,2], Miao Liu [1,2], Di Zhang [1,2], Bin Jiang [4], Xiaoying Li [5]

[1] Institute of Geriatrics, Chinese PLA General Hospital, 28 Fuxing Road, Beijing, 100853, China; [2] Beijing Key Laboratory of Aging and Geriatrics, Chinese PLA General Hospital, 28 Fuxing Road, Beijing, 100853, China; [3] State Key Laboratory of Kidney Disease, Chinese PLA General Hospital, 28 Fuxing Road, Beijing, 100853, China; [4] Department of Chinese Traditional Medicine and Acupuncture, Chinese PLA General Hospital, 28 Fuxing Road, Beijing, 100853, China; [5] Department of Geriatric Cardiology, Chinese PLA General Hospital, 28 Fuxing Road, Beijing, 100853, China; [6] Jinan Military Area CDC, Jinan, Shandong, China, 250014.

Figure legends

Supplementary Fig. S1. The LD plot of the 15 SNPs

Supplementary Table S1. Candidate SNPs from GWAS

Supplementary Table S2. Result of run tagger for LD plot

Supplementary Table S3. Effect of rs12914385 for smoking behavior in four models

Supplementary Table S4. Effect of rs2036534 for smoking behavior in four models

Supplementary Table S5. Effect of rs7937 for smoking behavior in four models

Supplementary Table S6. Effect of rs6474412 for smoking behavior in four models

Supplementary Table S7. Effect of rs6495308 for smoking behavior in four models

Supplementary Table S8. Effect of rs1329650 for smoking behavior in four models

Supplementary Table S9. Effect of rs4923457 for smoking behavior in four models

Supplementary Tables

Supplementary Table S1 Candidate SNPs from GWAS

SNP	Population	Gene	Chr	Alleles	Pmin	MAF	Reference
rs6474412	European	CHRNB3–CHRNA6	8	C/T	1.40E-08	0.221	[1]
rs13280604	European	CHRNB3–CHRNA6	8	G/A	1.30E-08	0.293	[1]
rs1451240	European, African American	CHRNB3	8	A/G	2.40E-08	0.293	[2]
rs4736835	European, African American	CHRNB3	8	C/T	3.00E-08	0.293	[2]
rs10958725	European, African American	CHRNB3	8	G/T	3.10E-08	0.329	[2]
rs1329650	European	LOC100188947	10	T/G	5.70E-10	0.317	[3]
rs1028936	European	LOC100188947	10	C/A	1.30E-09	0.390	[3]
rs4923457	European	BDNF	11	A/T	3.30E-08	0.420	[3]
rs6484320	European	BDNF	11	T/A	4.90E-08	0.367	[3]
rs879048	European	BDNF	11	C/A	4.90E-08	**0.011**	[3]
rs1051730	European	CHRNA3	15	G/A	2.80E-73	**0.037**	[1 3-5]
rs16969968	European	CHRNA3	15	G/A	5.60E-72	**0.037**	[3 4 6 7]
rs6495308	European	CHRNA3	15	C/T	5.80E-44	0.317	[4]
rs11638372	European	CHRNA5	15	C/T	1.0E-09	**0.035**	[5]
rs2036534	European	CHRNA5	15	C/T	4.00E-09	0.402	[5]
rs12914385	European	CHRNA5	15	C/T	4.20E-35	0.256	[3]
rs2036527	African American	CHRNA5	15	A/G	1.80E-08	**0.037**	[8]
rs8034191	European	AGPHD1	15	C/T	1.4E-63	**0.012**	[4]
rs11072768	European	CHRNB4	15	T/G	1.20E-26	0.159	[4]
rs7937	European	CYP2A6–CYP2B6	19	C/T	2.40E-09	0.341	[1]
rs3733829	European	EGLN2	19	G/A	1.00E-08	0.366	[3]

1. Thorgeirsson TE, Gudbjartsson DF, Surakka I, et al. Sequence variants at CHRNB3-CHRNA6 and CYP2A6 affect smoking behavior. Nature genetics 2010;**42**(5):448-53 doi: 10.1038/ng.573[published Online First: Epub Date]|.
2. Rice JP, Hartz SM, Agrawal A, et al. CHRNB3 is more strongly associated with Fagerstrom test for cigarette dependence-based nicotine dependence than cigarettes per day: phenotype definition changes genome-wide association studies results. Addiction (Abingdon, England) 2012;**107**(11):2019-28 doi: 10.1111/j.1360-0443.2012.03922.x[published Online First: Epub Date]|.
3. Consortium TaG. Genome-wide meta-analyses identify multiple loci associated with smoking behavior. Nature genetics 2010;**42**(5):441-7 doi: 10.1038/ng.571[published Online First: Epub Date]|.
4. Liu JZ, Tozzi F, Waterworth DM, et al. Meta-analysis and imputation refines the association of 15q25 with smoking quantity. Nature genetics 2010;**42**(5):436-40 doi: 10.1038/ng.572[published Online First: Epub Date]|.

5. Thorgeirsson TE, Geller F, Sulem P, et al. A variant associated with nicotine dependence, lung cancer and peripheral arterial disease. Nature 2008;**452**(7187):638-42 doi: 10.1038/nature06846[published Online First: Epub Date]|.

6. Saccone NL, Culverhouse RC, Schwantes-An TH, et al. Multiple independent loci at chromosome 15q25.1 affect smoking quantity: a meta-analysis and comparison with lung cancer and COPD. PLoS genetics 2010;**6**(8) doi: 10.1371/journal.pgen.1001053[published Online First: Epub Date]|.

7. Saccone SF, Saccone NL, Swan GE, et al. Systematic biological prioritization after a genome-wide association study: an application to nicotine dependence. Bioinformatics (Oxford, England) 2008;**24**(16):1805-11 doi: 10.1093/bioinformatics/btn315[published Online First: Epub Date]|.

8. David SP, Hamidovic A, Chen GK, et al. Genome-wide meta-analyses of smoking behaviors in African Americans. Translational psychiatry 2012;**2**:e119 doi: 10.1038/tp.2012.41[published Online First: Epub Date]|.

Supplementary Table S2 Result of run tagger for LD plot

Allele	Test	r^2
rs4923457	rs4923457	1
rs6484320	rs6484320	1
rs7937	rs7937	1
rs3733829	rs3733829	1
rs10958725	rs6474412	0.812
rs1451240	rs6474412	0.989
rs4736835	rs6474412	0.989
rs6474412	rs6474412	1
rs13280604	rs6474412	0.987
rs2036534	rs2036534	1
rs12914385	rs12914385	1
rs6495308	rs6495308	1
rs11072768	rs11072768	1
rs1329650	rs1329650	1
rs1028936	rs1028936	1

Supplementary Table S3. Effect of rs12914385 for smoking behavior in four models

	Case	rs12914385			Dominance model (CC+CT/TT)		Recessive model (CC/CT+TT)		Heterogeneous codominant model (CT/TT+CC)		Additive model (CC/CT/TT)	
	N	CC	CT	TT	P	OR	P	OR	P	OR	P	OR
Smoking[a]	1067	557 (28.8)	431 (31.0)	79(34.8)	0.105	1.26 (0.95-1.68)	0.077	1.14 (0.99-1.32)	0.320	1.08 (0.93-1.25)	**0.038**	**1.13 (1.01-1.27)**
Heavy smoking[a]	412	213 (11.0)	159 (11.4)	40 (17.6)	**0.003**	**1.70 (1.19-2.43)**	0.231	1.13 (0.92-1.39)	0.805	0.97 (0.79-1.20)	**0.033**	**1.19 (1.02-1.40)**
SI≤18[a]	777	404 (20.9)	311 (22.4)	62 (27.3)	**0.040**	**1.37 (1.01-1.86)**	0.118	1.14 (0.97-1.33)	0.572	1.05 (0.89-1.23)	**0.037**	**1.15 (1.01-1.30)**
Smoking[b]					0.199	1.25 (0.89-1.74)	0.141	1.14 (0.96-1.34)	0.396	1.08 (0.91-1.28)	0.089	1.13 (0.98-1.29)
Heavy smoking[b]					**0.009**	**1.70 (1.14-2.54)**	0.410	1.10 (0.88-1.37)	0.608	0.94 (0.75-1.18)	0.084	1.17 (0.98-1.40)
SI≤18[b]					0.075	1.36 (0.97-1.91)	0.250	1.11 (0.93-1.32)	0.791	1.03 (0.86-1.23)	0.100	1.13 (0.98-1.30)
Smoking[c]					0.198	1.25 (0.89-1.74)	0.146	1.13 (0.96-1.34)	0.407	1.08 (0.91-1.28)	0.092	1.12 (0.98-1.29)
Heavy smoking[c]					**0.010**	**1.69 (1.13-2.53)**	0.447	1.09 (0.87-1.36)	0.572	0.94 (0.74-1.18)	0.096	1.16 (0.97-1.39)
SI≤18[c]					0.078	1.36 (0.97-1.91)	0.254	1.11 (0.93-1.32)	0.792	1.03 (0.86-1.23)	0.103	1.13 (0.98-1.30)
Male												
Smoking[a]	832	429 (55.0)	343 (57.6)	60 (59.4)	0.519	1.15 (0.76-1.73)	0.276	1.12 (0.91-1.38)	0.437	1.09 (0.88-1.34)	0.255	1.10 (0.93-1.30)
Heavy smoking[a]	368	195 (25.0)	138 (23.2)	35 (34.7)	**0.020**	**1.66 (1.08-2.55)**	0.937	0.99 (0.78-1.25)	0.198	0.85 (0.67-1.09)	0.374	1.09 (0.90-1.32)
SI≤18[a]	622	319 (40.9)	255 (42.8)	48 (47.5)	0.255	1.27 (0.84-1.90)	0.317	1.11 (0.90-1.37)	0.667	1.05 (0.85-1.29)	0.205	1.11 (0.94-1.32)
Smoking[b]					0.619	1.11 (0.73-1.69)	0.180	1.16 (0.94-1.43)	0.267	1.13 (0.91-1.40)	0.200	1.12 (0.94-1.32)
Heavy smoking[b]					**0.028**	**1.63 (1.05-2.51)**	0.996	1.00 (0.79-1.28)	0.253	0.87 (0.68-1.11)	0.364	1.09 (0.90-1.32)
SI≤18[b]					0.279	1.25 (0.83-1.88)	0.277	1.12 (0.91-1.38)	0.585	1.06 (0.86-1.31)	0.189	1.12 (0.95-1.32)

Smoking[c]					0.621	1.11 (0.73-1.69)	0.179	1.16 (0.94-1.43)	0.266	1.13 (0.91-1.40)	0.200	1.12 (0.94-1.33)
Heavy smoking[c]					**0.031**	**1.62 (1.05-2.50)**	0.940	0.99 (1.78-1.26)	0.226	0.86 (0.67-1.10)	0.406	1.09 (0.90-1.31)
SI≤18[c]					0.287	1.25 (0.83-1.87)	0.297	1.12 (0.91-1.38)	0.610	1.06 (0.86-1.31)	0.203	1.12 (0.94-1.32)
Female												
Smoking[a]	235	128 (11.1)	88 (11.1)	19 (15.1)	0.171	1.43 (0.86-2.34)	0.702	1.06 (0.80-1.39)	0.776	0.96 (0.73-1.27)	0.399	1.10 (0.88-1.37)
Heavy smoking[a]	44	18 (1.6)	21 (2.6)	5 (4.0)	0.145	2.03 (0.78-5.23)	**0.050**	**1.84 (1.00-3.37)**	0.196	1.48 (0.82-2.70)	**0.029**	**1.65 (1.05-2.58)**
SI≤18[a]	155	85 (7.4)	56 (7.0)	14 (11.1)	0.111	1.60 (0.90-2.87)	0.836	1.04 (0.75-1.44)	0.564	1.05 (0.85-1.29)	0.425	1.11 (0.86-1.45)
Smoking[b]					0.164	1.46 (0.86-2.47)	0.623	1.07 (0.81-1.42)	0.853	0.97 (0.73-1.30)	0.340	1.12 (0.89-1.40)
Heavy smoking[b]					0.135	2.09 (0.80-5.46)	**0.040**	**1.90 (1.03-3.50)**	0.170	1.53 (0.83-2.79)	**0.023**	**1.69 (1.07-2.66)**
SI≤18[b]					0.108	1.63 (0.90-2.95)	0.781	1.05 (0.75-1.47)	0.609	0.91 (0.65-1.29)	0.389	1.13 (0.86-1.47)
Smoking[c]					0.158	1.46 (0.86-2.49)	0.629	1.07 (0.81-1.42)	0.838	0.97 (0.73-1.30)	0.339	1.12 (0.89-1.40)
Heavy smoking[c]					0.133	2.09 (0.80-5.48)	**0.038**	**1.91 (1.04-3.54)**	0.165	1.54 (0.84-2.82)	**0.022**	**1.70 (1.08-2.67)**
SI≤18[c]					0.108	1.63 (0.90-2.95)	0.766	1.05 (0.75-1.47)	0.620	0.92 (0.65-1.30)	0.380	1.13 (0.86-1.47)

a: Unadjusted

b:Adjusted for demographic characteristics (age, gender, education, occupation and ethnic)

c: Adjusted for demographic characteristics (age, gender, education, occupation and ethnic) , BMI and sports time

SI=age of smoking initiation

Supplementary Table S4. Effect of rs2036534 for smoking behavior in four models

	Cases	rs2036534			Dominance model (CC+CT/TT)		Recessive model (CC/CT+TT)		Heterogeneous codominant model (CT/TT+CC)		Additive model (CC/CT/TT)	
	N	CC	CT	TT	P	OR	P	OR	P	OR	P	OR
Smoking[a]	1067	173 (27.6)	540 (30.9)	354 (30.1)	0.967	1.00 (0.86-1.17)	0.142	1.16 (0.95-1.40)	0.280	1.08 (0.94-1.25)	0.415	1.04 (0.94-1.16)
Heavy smoking[a]	412	65 (10.4)	199 (11.4)	148 (12.6)	0.200	1.15 (0.93-1.43)	0.290	1.16 (0.88-1.54)	0.690	0.96 (0.78-1.18)	0.148	1.12 (0.96-1.30)
SI≤18[a]	777	125 (19.9)	387 (22.1)	265 (22.5)	0.512	1.06 (0.89-1.25)	0.197	1.15 (0.93-1.43)	0.714	1.03 (0.88-1.21)	0.250	1.07 (0.95-1.20)
Smoking[b]					0.649	1.04 (0.87-1.25)	0.119	1.20 (0.96-1.50)	0.445	1.07 (0.90-1.26)	0.251	1.07 (0.95-1.21)
Heavy smoking[b]					0.107	1.21 (0.96-1.53)	0.283	1.18 (0.87-1.60)	0.486	0.92 (0.74-1.16)	0.093	1.15 (0.98-1.35)
SI≤18[b]					0.343	1.09 (0.91-1.32)	0.206	1.17 (0.92-1.47)	0.943	1.01 (0.84-1.20)	0.182	1.09 (0.96-1.24)
Smoking[c]					0.650	1.04 (0.87-1.25)	0.119	1.20 (0.96-1.50)	0.445	1.07 (0.90-1.26)	0.252	1.07 (0.95-1.21)
Heavy smoking[c]					0.128	1.20 (0.95-1.52)	0.284	1.18 (0.87-1.60)	0.540	0.93 (0.75-1.17)	0.106	1.14 (0.97-1.35)
SI≤18[c]					0.362	1.09 (0.91-1.31)	0.196	1.17 (0.92-1.48)	0.898	1.01 (0.85-1.21)	0.185	1.09 (0.96-1.24)
Male												
Smoking[a]	832	143 (55.9)	415 (55.9)	274 (56.0)	0.871	0.98 (0.80-1.22)	0.867	1.02 (0.78-1.34)	0.780	1.03 (0.84-1.27)	0.985	1.00 (0.86-1.16)
Heavy smoking[a]	368	61 (23.8)	173 (23.6)	134 (27.4)	0.120	1.22 (0.95-1.56)	0.658	1.07 (0.78-1.47)	0.259	0.87 (0.69-1.11)	0.194	1.12 (0.94-1.33)
SI≤18[a]	622	106 (41.4)	308 (42.1)	208 (42.5)	0.817	1.03 (0.82-1.28)	0.801	1.04 (0.79-1.36)	0.978	1.00 (0.81-1.23)	0.768	1.02 (0.88-1.19)
Smoking[b]					0.998	1.00 (0.80-1.25)	0.841	1.03 (0.78-1.36)	0.877	1.02 (0.82-1.25)	0.927	1.01 (0.87-1.17)
Heavy smoking[b]					0.085	1.25 (0.97-1.60)	0.589	1.09 (0.79-1.50)	0.226	0.86 (0.68-1.10)	0.143	1.14 (0.96-1.36)
SI≤18[b]					0.778	1.03 (0.83-1.29)	0.794	1.04 (0.79-1.36)	0.946	0.99 (0.81-1.22)	0.738	1.03 (0.88-1.19)

Smoking[c]					0.992	1.00 (0.80-1.25)	0.837	1.03 (0.78-1.36)	0.868	1.02 (0.83-1.26)	0.928	1.01 (0.87-1.17)
Heavy smoking[c]					0.105	1.23 (0.96-1.58)	0.599	1.09 (0.79-1.50)	0.261	0.87 (0.69-1.11)	0.166	1.13 (0.95-1.35)
SI≤18[c]					0.827	1.03 (0.82-1.28)	0.793	1.04 (0.79-1.37)	0.994	1.00 (0.81-1.23)	0.771	1.02 (0.88-1.19)
Female												
Smoking[a]	235	30 (8.1)	125 (12.3)	80 (11.6)	0.755	1.05 (0.79-1.39)	**0.031**	**1.55 (1.04-2.32)**	0.172	1.21 (0.92-1.59)	0.165	1.15 (0.94-1.40)
Heavy smoking[a]	44	4 (1.1)	26 (2.6)	14 (2.0)	0.851	0.94 (0.50-1.79)	0.134	2.20 (0.78-6.20)	0.178	1.52 (0.83-2.79)	0.474	1.17 (0.76-1.81)
SI≤18[a]	155	19 (5.1)	79 (7.8)	57 (8.3)	0.318	1.19 (0.85-1.67)	0.060	1.61 (0.98-2.63)	0.608	1.09 (0.79-1.51)	0.087	1.23 (0.97-1.57)
Smoking[b]					0.651	1.07 (0.80-1.44)	**0.018**	**1.64 (1.09-2.47)**	0.158	1.22 (0.92-1.62)	0.102	1.18 (0.97-1.45)
Heavy smoking[b]					0.919	0.97 (0.51-1.85)	0.116	2.30 (0.81-6.49)	0.181	1.52 (0.82-2.80)	0.410	1.20 (0.78-1.86)
SI≤18[b]					0.265	1.22 (0.86-1.72)	**0.045**	**1.67 (1.01-2.74)**	0.609	1.09 (0.78-1.52)	0.063	1.26 (0.99-1.61)
Smoking[c]					0.620	1.08 (0.80-1.45)	**0.017**	**1.65 (1.10-2.50)**	0.162	1.22 (0.92-1.62)	0.092	1.19 (0.97-1.46)
Heavy smoking[c]					0.940	0.98 (0.51-1.86)	0.110	2.33 (0.82-6.60)	0.182	1.52 (0.82-2.80)	0.390	1.21 (0.78-1.88)
SI≤18[c]					0.257	1.22 (0.86-1.73)	**0.042**	**1.68 (1.02-2.77)**	0.606	1.09 (0.78-1.52)	0.058	1.27 (0.99-1.61)

a: Unadjusted

b:Adjusted for demographic characteristics (age, gender, education, occupation and ethnic)

c: Adjusted for demographic characteristics (age, gender, education, occupation and ethnic) , BMI and sports time

SI=age of smoking initiation

Supplementary Table S5. Effect of rs7937 for smoking behavior in four models

	Cases	rs7937			Dominance model (CC+CT/TT)		Recessive model (CC/CT+TT)		Heterogeneous codominant model (CT/TT+CC)		Additive model (CC/CT/TT)	
	N	CC	CT	TT	P	OR	P	OR	P	OR	P	OR
Smoking[a]	1067	99 (26.4)	467 (30.4)	501 (30.5)	0.533	1.05 (0.91-1.21)	0.105	1.22 (0.96-1.56)	0.705	1.03 (0.89-1.19)	0.219	1.07 (0.96-1.19)
Heavy smoking[a]	412	32 (8.5)	180 (11.7)	200 (12.2)	0.302	1.11 (0.91-1.37)	0.051	1.46 (1.00-2.12)	0.861	1.02 (0.83-1.25)	0.093	1.15 (0.98-1.34)
SI≤18[a]	777	72 (19.2)	340 (22.1)	365 (22.3)	0.605	1.04 (0.89-1.22)	0.187	1.20 (0.92-1.57)	0.765	1.03 (0.87-1.20)	0.317	1.06 (0.94-1.20)
Smoking[b]					0.305	1.09 (0.92-1.29)	0.165	1.22 (0.92-1.62)	0.856	0.98 (0.83-1.17)	0.150	1.10 (0.97-1.25)
Heavy smoking[b]					0.155	1.18 (0.94-1.47)	0.085	1.43 (0.95-2.14)	0.700	0.96 (0.76-1.20)	0.062	1.18 (0.99-1.40)
SI≤18[b]					0.409	1.08 (0.90-1.29)	0.364	1.15 (0.85-1.55)	0.781	0.98 (0.82-1.17)	0.297	1.07 (0.94-1.23)
Smoking[c]					0.300	1.09 (0.92-1.29)	0.161	1.23 (0.92-1.63)	0.855	0.98 (0.83-1.17)	0.146	1.10 (0.97-1.25)
Heavy smoking[c]					0.154	1.18 (0.94-1.47)	0.085	1.43 (0.95-2.14)	0.698	0.96 (0.76-1.20)	0.061	1.18 (0.99-1.40)
SI≤18[c]					0.420	1.08 (0.90-1.28)	0.366	1.15 (0.85-1.55)	0.793	0.98 (0.82-1.17)	0.305	1.07 (0.94-1.23)
Male												
Smoking[a]	832	71 (50.7)	364 (54.8)	397 (59.0)	0.060	1.22 (0.99-1.50)	0.160	1.28 (0.91-1.82)	0.290	0.89 (0.73-1.10)	**0.037**	**1.18 (1.01-1.39)**
Heavy smoking[a]	368	30 (21.4)	160 (24.1)	178 (26.4)	0.213	1.16 (0.92-1.47)	0.317	1.24 (0.81-1.89)	0.511	0.92 (0.73-1.17)	0.159	1.14 (0.95-1.37)
SI≤18[a]	622	53 (37.9)	271 (40.8)	298 (44.3)	0.123	1.18 (0.96-1.45)	0.284	1.22 (0.85-1.74)	0.361	0.91 (0.74-1.12)	0.095	1.15 (0.98-1.35)
Smoking[b]					**0.048**	**1.24 (1.00-1.53)**	0.082	1.37 (0.96-1.96)	0.339	0.90 (0.73-1.11)	**0.021**	**1.21 (1.03-1.42)**
Heavy					0.172	1.18 (0.93-1.50)	0.241	1.29 (0.84-1.98)	0.502	0.92 (0.72-1.17)	0.115	1.16 (0.96-1.40)

smoking[b]												
SI≤18[b]					0.116	1.18 (0.96-1.46)	0.238	1.24 (0.87-1.78)	0.379	0.91 (0.74-1.12)	0.082	1.15 (0.98-1.35)
Smoking[c]					**0.049**	**1.24 (1.00-1.53)**	0.082	1.37 (0.96-1.96)	0.341	0.90 (0.73-1.12)	**0.021**	**1.21 (1.03-1.42)**
Heavy smoking[c]					0.169	1.18 (0.93-1.51)	0.239	1.29 (0.84-1.99)	0.498	0.92 (0.72-1.17)	0.112	1.16 (0.97-1.40)
SI≤18[c]					0.115	1.18 (0.96-1.46)	0.235	1.24 (0.87-1.78)	0.381	0.91 (0.74-1.12)	0.081	1.15 (0.98-1.36)
Female												
Smoking[a]	235	28 (11.9)	103 (11.8)	104 (10.8)	0.448	0.90 (0.69-1.18)	0.760	0.94 (0.62-1.43)	0.569	1.08 (0.82-1.42)	0.486	0.93 (0.76-1.14)
Heavy smoking[a]	44	2 (0.9)	20 (2.3)	22 (2.3)	0.646	1.15 (0.63-2.09)	0.169	2.72 (0.65-11.31)	0.649	1.15 (0.63-2.09)	0.312	1.27 (0.80-2.03)
SI≤18[a]	155	19 (8.1)	69 (7.9)	67 (6.9)	0.384	0.86 (0.62-1.20)	0.702	0.91 (0.55-1.50)	0.527	1.11 (0.80-1.55)	0.410	0.90 (0.71-1.15)
Smoking[b]					0.380	0.88 (0.67-1.17)	0.966	1.01 (0.66-1.56)	0.358	1.14 (0.86-1.52)	0.571	0.94 (0.77-1.16)
Heavy smoking[b]					0.690	1.13 (0.62-2.06)	0.130	3.02 (0.722-12.60)	0.513	1.22 (0.67-2.24)	0.296	1.28 (0.81-2.03)
SI≤18[b]					0.343	0.85 (0.61-1.19)	0.892	0.97 (0.58-1.61)	0.382	1.16 (0.83-1.62)	0.448	0.91 (0.72-1.16)
Smoking[c]					0.379	0.88 (0.66-1.17)	0.932	1.02 (0.66-1.57)	0.343	1.14 (0.86-1.52)	0.582	0.94 (0.77-1.16)
Heavy smoking[c]					0.698	1.13 (0.62-2.06)	0.127	3.05 (0.73-12.73)	0.498	1.23 (0.67-2.26)	0.296	1.28 (0.81-2.03)
SI≤18[c]					0.336	0.85 (0.61-1.19)	0.893	0.97 (0.58-1.61)	0.374	1.16 (0.83-1.63)	0.442	0.91 (0.72-1.16)

a: Unadjusted

b:Adjusted for demographic characteristics (age, gender, education, occupation and ethnic)

c: Adjusted for demographic characteristics (age, gender, education, occupation and ethnic) , BMI and sports time

SI=age of smoking initiation

Supplementary Table S6. Effect of rs6474412 for smoking behavior in four models

	Cases	rs6474412			Dominance model (CC+CT/TT)		Recessive model (CC/CT+TT)		Heterogeneous codominant model (CT/TT+CC)		Additive model (CC/CT/TT)	
	N	CC	CT	TT	P	OR	P	OR	P	OR	P	OR
Smoking[a]	1067	45 (27.8)	356 (30.6)	666 (29.9)	0.815	0.98 (0.85-1.14)	0.522	1.12 (0.79-1.59)	0.599	1.04 (0.89-1.21)	0.981	1.00 (0.89-1.13)
Heavy smoking[a]	412	22 (13.6)	137 (11.8)	253 (11.4)	0.562	0.94 (0.76-1.16)	0.420	0.83 (0.52-1.31)	0.811	1.03 (0.83-1.28)	0.438	0.93 (0.78-1.11)
SI≤18[a]	777	25 (15.4)	254 (21.8)	498 (22.4)	0.366	1.08 (0.92-1.27)	**0.044**	**1.56 (1.01-2.41)**	0.977	1.00 (0.84-1.18)	0.137	1.11 (0.97-1.28)
Smoking[b]					0.855	1.02 (0.85-1.21)	0.418	1.18 (0.79-1.77)	0.861	1.02 (0.85-1.22)	0.670	1.03 (0.89-1.19)
Heavy smoking[b]					0.764	0.97 (0.77-1.21)	0.457	0.83 (0.50-1.37)	0.980	1.00 (0.79-1.26)	0.602	0.95 (0.79-1.15)
SI≤18[b]					0.151	1.14 (0.95-1.37)	**0.022**	**1.72 (1.08-2.75)**	0.655	0.96 (0.79-1.16)	**0.042**	**1.17 (1.01-1.37)**
Smoking[c]					0.867	1.02 (0.85-1.21)	0.421	1.18 (0.79-1.77)	0.850	1.02 (0.85-1.22)	0.679	1.03 (0.89-1.19)
Heavy smoking[c]					0.764	0.97 (0.77-1.21)	0.434	0.82 (0.49-1.35)	0.967	1.00 (0.79-1.26)	0.593	0.95 (0.79-1.15)
SI≤18[c]					0.150	1.14 (0.95-1.37)	**0.023**	**1.72 (1.08-2.73)**	0.647	0.96 (0.79-1.15)	**0.042**	**1.17 (1.01-1.37)**
Male												
Smoking[a]	832	35 (50.0)	285 (57.1)	512 (56.4)	0.955	1.01 (0.81-1.24)	0.275	1.31 (0.81-2.11)	0.664	1.05 (0.84-1.30)	0.656	1.04 (0.87-1.24)
Heavy smoking[a]	368	19 (27.1)	124 (24.8)	225 (24.8)	0.879	0.98 (0.77-1.25)	0.659	0.89 (0.52-1.52)	0.967	1.00 (0.78-1.28)	0.773	0.97 (0.79-1.19)
SI≤18[a]	622	20 (28.6)	208 (41.7)	394 (43.4)	0.208	1.15 (0.93-1.42)	**0.020**	**1.87 (1.10-3.17)**	0.811	0.97 (0.78-1.21)	0.057	1.19 (1.00-1.42)
Smoking[b]					0.872	0.98 (0.79-1.22)	0.325	1.28 (0.79-2.08)	0.541	1.07 (0.86-1.34)	0.833	1.02 (0.85-1.22)
Heavy					0.772	0.96 (0.75-1.23)	0.660	0.88 (0.51-1.53)	0.920	1.01 (0.79-1.31)	0.688	0.96 (0.78-1.18)

smoking[b]												
SI≤18[b]					0.236	1.14 (0.92-1.41)	**0.023**	**1.85 (1.09-3.15)**	0.853	0.98 (0.79-1.22)	0.068	1.18 (0.99-1.42)
Smoking[c]					0.874	0.98 (0.79-1.22)	0.327	1.28 (0.78-2.08)	0.544	1.07 (0.86-1.34)	0.832	1.02 (0.85-1.22)
Heavy smoking[c]					0.779	0.97 (0.76-1.24)	0.632	0.88 (0.51-1.51)	0.942	1.01 (0.78-1.30)	0.683	0.96 (0.78-1.18)
SI≤18[c]					0.232	1.14 (0.92-1.41)	**0.024**	**1.84 (1.08-3.13)**	0.838	0.98 (0.79-1.22)	0.068	1.18 (0.99-1.42)
Female												
Smoking[a]	235	10 (10.9)	71 (10.7)	154 (11.7)	0.510	1.10 (0.83-1.46)	0.889	1.05 (0.54-2.05)	0.536	0.91 (0.68-1.22)	0.553	1.08 (0.85-1.37)
Heavy smoking[a]	44	3 (3.3)	13 (2.0)	28 (2.1)	0.994	1.00 (0.54-1.57)	0.441	0.63 (0.19-2.06)	0.726	0.89 (0.46-1.71)	0.785	0.93 (0.56-1.55)
SI≤18[a]	155	5 (5.4)	46 (6.9)	104 (7.9)	0.345	1.18 (0.84-1.67)	0.451	1.42 (0.57-3.56)	0.522	0.89 (0.62-1.27)	0.288	1.18 (0.87-1.58)
Smoking[b]					0.555	1.09 (0.81-1.47)	0.970	0.99 (0.49-1.97)	0.533	0.91 (0.67-1.23)	0.643	1.06 (0.83-1.36)
Heavy smoking[b]					0.979	0.99 (0.53-1.85)	0.394	0.59 (0.18-1.98)	0.729	0.89 (0.46-1.72)	0.744	0.92 (0.55-1.54)
SI≤18[b]					0.367	1.18 (0.83-1.67)	0.511	1.37 (0.54-3.45)	0.519	0.89 (0.62-1.28)	0.321	1.17 (0.86-1.58)
Smoking[c]					0.590	1.08 (0.81-1.46)	0.963	0.98 (0.49-1.97)	0.565	0.91 (0.67-1.24)	0.675	1.06 (0.82-1.35)
Heavy smoking[c]					0.947	0.98 (0.52-1.83)	0.392	0.59 (0.18-1.98)	0.759	0.90 (0.47-1.75)	0.718	0.91 (0.54-1.52)
SI≤18[c]					0.375	1.17 (0.82-1.67)	0.512	1.36 (0.54-3.45)	0.529	0.89 (0.62-1.28)	0.328	1.16 (0.86-1.58)

a: Unadjusted

b:Adjusted for demographic characteristics (age, gender, education, occupation and ethnic)

c: Adjusted for demographic characteristics (age, gender, education, occupation and ethnic) , BMI and sports time

SI=age of smoking initiation

Supplementary Table S7. Effect of rs6495308 for smoking behavior in four models

	Cases	rs6495308			Dominance model (TT+CT/CC)		Recessive model (TT/CT+CC)		Heterogeneous codominant model (CT/TT+CC)		Additive model (TT/CT/CC)	
	N	TT	CT	CC	P	OR	P	OR	P	OR	P	OR
Smoking[a]	1067	83 (26.3)	445 (30.6)	539 (30.3)	0.745	1.02 (0.89-1.18)	0.118	1.23 (0.95-1.60)	0.564	1.04 (0.90-1.21)	0.342	1.06 (0.94-1.18)
Heavy smoking[a]	412	30 (9.5)	179 (12.3)	203 (11.4)	0.721	0.96 (0.78-1.18)	0.215	1.28 (0.87-1.89)	0.279	1.12 (0.91-1.38)	0.786	1.02 (0.87-1.20)
SI≤18[a]	777	63 (19.9)	309 (21.2)	405 (22.8)	0.202	1.11 (0.95-1.30)	0.368	1.141 (0.86-1.52)	0.437	0.94 (0.80-1.10)	0.168	1.09 (0.96-1.24)
Smoking[b]					0.847	0.98 (0.83-1.16)	0.219	1.21 (0.89-1.64)	0.365	1.08 (0.91-1.28)	0.697	1.03 (0.90-1.17)
Heavy smoking[b]					0.484	0.92 (0.74-1.15)	0.384	1.21 (0.79-1.83)	0.229	1.15 (0.92-1.44)	0.867	0.99 (0.83-1.17)
SI≤18[b]					0.320	1.09 (0.92-1.30)	0.552	1.10 (0.80-1.51)	0.502	0.94 (0.79-1.13)	0.303	1.08 (0.94-1.23)
Smoking[c]					0.846	0.98 (0.83-1.16)	0.222	1.21 (0.89-1.64)	0.367	1.08 (0.91-1.28)	0.700	1.03 (0.90-1.17)
Heavy smoking[c]					0.497	0.93 (0.74-1.16)	0.405	1.20 (0.79-1.82)	0.246	1.14 (0.91-1.43)	0.866	0.99 (0.83-1.17)
SI≤18[c]					0.315	1.10 (0.92-1.31)	0.570	1.10 (0.80-1.51)	0.485	0.94 (0.78-1.12)	0.305	1.07 (0.94-1.23)
Male												
Smoking[a]	832	64 (50.4)	338 (56.4)	430 (57.3)	0.465	1.08 (0.88-1.33)	0.159	1.30 (0.90-1.87)	0.951	1.01 (0.82-1.24)	0.238	1.10 (0.94-1.29)
Heavy smoking[a]	368	27 (21.3)	162 (27.0)	179 (23.8)	0.329	0.89 (0.70-1.13)	0.320	1.25 (0.80-1.95)	0.118	1.21 (0.95-1.54)	0.746	0.97 (0.81-1.16)
SI≤18[a]	622	50 (39.4)	237 (39.6)	335 (44.6)	**0.048**	**1.23 (1.00-1.52)**	0.513	1.13 (0.78-1.64)	0.102	0.84 (0.68-1.04)	0.070	1.16 (0.99-1.37)
Smoking[b]					0.501	1.08 (0.87-1.33)	0.244	1.25 (0.86-1.81)	0.986	1.00 (0.81-1.24)	0.302	1.09 (0.93-1.28)
Heavy					0.311	0.88 (0.70-1.12)	0.458	1.19 (0.76-1.86)	0.147	1.20 (0.94-1.52)	0.639	0.96 (0.79-1.15)

smoking[b]												
SI≤18[b]					**0.050**	**1.23 (1.00-1.51)**	0.575	1.11 (0.77-1.62)	0.094	0.84 (0.68-1.03)	0.078	1.16 (0.98-1.36)
Smoking[c]					0.503	1.07 (0.87-1.33)	0.246	1.25 (0.86-1.81)	0.987	1.00 (0.81-1.24)	0.304	1.09 (0.93-1.28)
Heavy smoking[c]					0.320	0.89 (0.70-1.13)	0.478	1.18 (0.75-1.84)	0.158	1.19 (0.93-1.52)	0.638	0.96 (0.79-1.15)
SI≤18[c]					**0.050**	**1.23 (1.00-1.52)**	0.592	1.11 (0.76-1.61)	0.090	0.83 (0.67-1.03)	0.079	1.16 (0.98-1.36)
Female												
Smoking[a]	235	19 (10.0)	107 (12.5)	109 (10.6)	0.300	0.87 (0.66-1.14)	0.547	1.16 (0.71-1.91)	0.160	1.22 (0.93-1.60)	0.602	0.95 (0.77-1.16)
Heavy smoking[a]	44	3 (1.6)	17 (2.0)	24 (2.3)	0.505	1.23 (0.67-2.23)	0.589	1.39 (0.43-4.52)	0.719	0.89 (0.48-1.65)	0.452	1.20 (0.75-1.94)
SI≤18[a]	155	13 (6.8)	72 (8.4)	70 (6.8)	0.255	0.83 (0.60-1.15)	0.731	1.11 (0.62-2.00)	0.175	1.26 (0.90-1.74)	0.469	0.91 (0.71-1.17)
Smoking[b]					0.245	0.85 (0.64-1.12)	0.596	1.15 (0.69-1.91)	0.136	1.24 (0.94-1.64)	0.495	0.93 (0.75-1.15)
Heavy smoking[b]					0.519	1.22 (0.67-2.24)	0.611	1.36 (0.41-4.47)	0.719	0.89 (0.48-1.66)	0.471	1.19 (0.74-1.93)
SI≤18[b]					0.221	0.81 (0.58-1.13)	0.782	1.09 (0.60-1.98)	0.160	1.27 (0.91-1.78)	0.411	0.90 (0.70-1.16)
Smoking[c]					0.211	0.84 (0.63-1.11)	0.609	1.14 (0.69-1.90)	0.117	1.25 (0.95-1.67)	0.450	0.92 (0.74-1.14)
Heavy smoking[c]					0.541	1.21 (0.66-2.22)	0.617	1.35 (0.41-4.45)	0.741	0.90 (0.49-1.68)	0.489	1.19 (0.73-1.92)
SI≤18[c]					0.216	0.81 (0.58-1.13)	0.788	1.09 (0.60-1.97)	0.158	1.27 (0.91-1.78)	0.404	0.90 (0.70-1.16)

a: Unadjusted

b:Adjusted for demographic characteristics (age, gender, education, occupation and ethnic)

c: Adjusted for demographic characteristics (age, gender, education, occupation and ethnic) , BMI and sports time

SI=age of smoking initiation

Supplementary Table S8. Effect of rs1329650 for smoking behavior in four models

	Cases	rs1329650			Dominance model (AA+CA)/CC		Recessive model AA/(CA+CC)		Heterogeneous codominant model CA/(AA+CC)		Additive model AA/CA/CC	
	N	AA	CA	CC	P	OR	P	OR	P	OR	P	OR
Smoking[a]	1067	532 (29.3)	430 (30.7)	105 (31.3)	0.582	1.07 (0.84-1.37)	0.317	1.08 (0.93-1.24)	0.488	1.05 (0.91-1.22)	0.311	1.06 (0.95-1.18)
Heavy smoking[a]	412	222 (12.2)	161 (11.5)	29 (8.7)	0.079	0.70 (0.47-1.04)	0.236	0.88 (0.72-1.09)	0.876	0.98 (0.80-1.21)	0.092	0.87 (0.74-1.02)
SI≤18[a]	777	396 (21.8)	299 (21.3)	82 (24.5)	0.225	1.18 (0.91-1.53)	0.912	1.01 (0.86-1.18)	0.540	0.95 (0.81-1.12)	0.533	1.04 (0.92-1.17)
Smoking[b]					0.681	1.06 (0.80-1.41)	0.114	1.15 (0.97-1.36)	0.170	1.13 (0.95-1.34)	0.178	1.09 (0.96-1.24)
Heavy smoking[b]					**0.028**	**0.62 (0.41-0.95)**	0.298	0.89 (0.71-1.11)	0.785	1.03 (0.82-1.30)	0.076	0.86 (0.72-1.02)
SI≤18[b]					0.255	1.19 (0.89-1.59)	0.639	1.04 (0.88-1.24)	0.837	0.98 (0.82-1.18)	0.388	1.06 (0.93-1.21)
Smoking[c]					0.682	1.06 (0.80-1.41)	0.114	1.15 (0.97-1.36)	0.170	1.13 (0.95-1.34)	0.179	1.09 (0.96-1.24)
Heavy smoking[c]					**0.029**	**0.63 (0.41-0.95)**	0.279	0.88 (0.71-1.11)	0.825	1.03 (0.82-1.29)	0.072	0.85 (0.72-1.01)
SI≤18[c]					0.249	1.19 (0.89-1.59)	0.647	1.04 (0.87-1.24)	0.821	0.98 (0.82-1.17)	0.389	1.06 (0.93-1.21)
Male												
Smoking[a]	832	403 (52.5)	346 (61.1)	83 (57.6)	0.739	1.06 (0.75-1.50)	**0.002**	**1.38 (1.12-1.70)**	**0.003**	**1.38 (1.11-1.70)**	**0.014**	**1.22 (1.04-1.42)**
Heavy smoking[a]	368	193 (25.2)	149 (26.3)	26 (18.1)	**0.047**	**0.64 (0.41-0.99)**	0.819	0.97 (0.77-1.23)	0.324	1.13 (0.89-1.44)	0.285	0.91 (0.76-1.09)
SI≤18[a]	622	314 (40.9)	241 (42.6)	67 (46.5)	0.259	1.22 (0.86-1.72)	0.342	1.11 (0.90-1.36)	0.774	1.03 (0.83-1.28)	0.222	1.10 (0.94-1.29)
Smoking[b]					0.710	1.07 (0.75-1.53)	**0.002**	**1.39 (1.13-1.72)**	**0.004**	**1.38 (1.11-1.72)**	**0.014**	**1.22 (1.04-1.43)**

Heavy smoking[b]					**0.031**	**0.61 (0.39-0.96)**	0.760	0.96 (0.76-1.22)	0.312	1.14 (0.89-1.45)	0.230	0.89 (0.74-1.07)
SI≤18[b]					0.252	1.22 (0.87-1.73)	0.352	1.10 (0.90-1.36)	0.796	1.03 (0.83-1.27)	0.224	1.10 (0.94-1.29)
Smoking[c]					0.708	1.07 (0.75-1.53)	**0.002**	**1.39 (1.13-1.72)**	**0.004**	**1.38 (1.11-1.72)**	**0.014**	**1.22 (1.04-1.43)**
Heavy smoking[c]					**0.032**	**0.61 (0.39-0.96)**	0.724	0.96 (0.75-1.22)	0.340	1.13 (0.88-1.44)	0.218	0.89 (0.74-1.07)
SI≤18[c]					0.248	1.23 (0.87-1.74)	0.366	1.10 (0.89-1.35)	0.822	1.03 (0.83-1.27)	0.230	1.10 (0.94-1.29)
Female												
Smoking[a]	235	129 (12.3)	84 (10.1)	22 (11.5)	0.928	1.02 (0.64-1.63)	0.160	0.82 (0.63-1.08)	0.128	0.81 (0.61-1.07)	0.303	0.90 (0.72-1.11)
Heavy smoking[a]	44	29 (2.8)	12 (1.4)	3 (1.6)	0.582	0.72 (0.22-2.34)	**0.043**	**0.52 (0.28-0.98)**	0.081	0.55 (0.28-1.08)	0.072	0.62 (0.37-1.04)
SI≤18[a]	155	82 (7.8)	58 (6.9)	15 (7.9)	0.831	1.06 (0.61-1.85)	0.547	0.90 (0.65-1.26)	0.450	0.88 (0.63-1.23)	0.714	0.95 (0.74-1.23)
Smoking[b]					0.840	1.05 (0.65-1.70)	0.134	0.81 (0.61-1.07)	0.100	0.78 (0.59-1.05)	0.276	0.89 (0.71-1.10)
Heavy smoking[b]					0.584	0.72 (0.22-2.36)	**0.037**	**0.51 (0.27-0.96)**	0.070	0.54 (0.27-1.05)	0.064	0.61 (0.36-1.03)
SI≤18[b]					0.769	1.09 (0.62-1.91)	0.523	0.90 (0.64-1.25)	0.411	0.87 (0.61-1.22)	0.718	0.95 (0.74-1.23)
Smoking[c]					0.863	1.04 (0.64-1.69)	0.136	0.81 (0.61-1.07)	0.105	0.79 (0.59-1.05)	0.272	0.88 (0.71-1.10)
Heavy smoking[c]					0.572	0.71 (0.22-2.33)	**0.038**	**0.51 (0.27-0.96)**	0.073	0.54 (0.28-1.06)	0.064	0.61 (0.36-1.03)
SI≤18[c]					0.771	1.09 (0.62-1.91)	0.527	0.90 (0.64-1.25)	0.415	0.87 (0.62-1.22)	0.721	0.95 (0.74-1.23)

a: Unadjusted

b:Adjusted for demographic characteristics (age, gender, education, occupation and ethnic)

c: Adjusted for demographic characteristics (age, gender, education, occupation and ethnic) , BMI and sports time

SI=age of smoking initiation

Supplementary Table S9. Effect of rs4923457 for smoking behavior in four models

	Cases	rs4923457			Dominance model (TT+AT/AA)		Recessive model (TT/AT+AA)		Heterogeneous codominant model (AT/TT+AA)		Additive model (TT/AT/AA)	
	N	TT	AT	AA	P	OR	P	OR	P	OR	P	OR
Smoking[a]	1067	191 (30.0)	538 (30.0)	338 (30.1)	0.953	1.01 (0.86-1.17)	0.977	1.00 (0.83-1.21)	0.973	1.00 (0.86-1.15)	0.957	1.00 (0.90-1.11)
Heavy smoking[a]	412	77 (12.1)	196 (10.9)	139 (12.4)	0.323	1.12 (0.90-1.39)	0.669	0.94 (0.73-1.23)	0.212	0.88 (0.71-1.08)	0.668	1.03 (0.89-1.20)
SI≤18[a]	777	138 (21.7)	399 (22.3)	240 (21.4)	0.626	0.96 (0.81-1.14)	0.890	1.02 (0.82-1.25)	0.576	1.05 (0.89-1.23)	0.801	0.99 (0.88-1.11)
Smoking[b]					0.995	1.00 (0.84-1.20)	0.681	1.05 (0.84-1.30)	0.757	1.03 (0.87-1.22)	0.825	1.01 (0.90-1.15)
Heavy smoking[b]					0.361	1.12 (0.88-1.42)	0.848	0.97 (0.73-1.30)	0.318	0.89 (0.71-1.12)	0.614	1.04 (0.89-1.22)
SI≤18[b]					0.585	0.95 (0.78-1.15)	0.670	1.05 (0.84-1.32)	0.403	1.08 (0.90-1.29)	0.897	0.99 (0.87-1.13)
Smoking[c]					0.990	1.00 (0.84-1.20)	0.686	1.05 (0.84-1.30)	0.764	1.03 (0.87-1.21)	0.825	1.01 (0.90-1.15)
Heavy smoking[c]					0.364	1.12 (0.88-1.42)	0.968	0.97 (0.73-1.29)	0.310	0.89 (0.71-1.11)	0.626	1.04 (0.89-1.22)
SI≤18[c]					0.585	0.95 (0.78-1.15)	0.688	1.05 (0.83-1.32)	0.414	1.08 (0.90-1.28)	0.886	0.99 (0.87-1.13)
Male												
Smoking[a]	832	148 (54.8)	410 (55.3)	274 (58.9)	0.173	1.17 (0.93-1.46)	0.579	1.08 (0.83-1.41)	0.403	0.92 (0.75-1.13)	0.221	1.10 (0.95-1.27)
Heavy smoking[a]	368	64 (23.7)	170 (22.9)	134 (28.8)	**0.019**	**1.35 (1.05-1.73)**	0.611	1.08 (0.80-1.48)	0.074	0.81 (0.64-1.02)	0.063	1.18 (0.99-1.40)
SI≤18[a]	622	109 (40.4)	314 (42.3)	199 (42.8)	0.718	1.04 (0.83-1.30)	0.521	1.09 (0.84-1.43)	0.872	1.02 (0.83-1.25)	0.549	1.05 (0.90-1.22)
Smoking[b]					0.235	1.15 (0.92-1.44)	0.518	1.09 (0.83-1.43)	0.547	0.94 (0.76-1.16)	0.253	1.09 (0.94-1.27)
Heavy					**0.032**	**1.32 (1.02-1.70)**	0.556	1.10 (0.80-1.50)	0.123	0.83 (0.65-1.05)	0.078	1.17 (0.98-1.39)

smoking[b]												
SI≤18[b]					0.774	1.03 (0.83-1.29)	0.509	1.09 (0.84-1.43)	0.806	1.03 (0.83-1.26)	0.575	1.04 (0.90-1.21)
Smoking[c]					0.233	1.15 (0.92-1.44)	0.518	1.09 (0.83-1.43)	0.544	0.94 (0.76-1.16)	0.252	1.09 (0.94-1.27)
Heavy smoking[c]					**0.031**	**1.32 (1.03-1.70)**	0.564	1.10 (0.80-1.50)	0.118	0.83 (0.65-1.05)	0.078	1.17 (0.98-1.39)
SI≤18[c]					0.761	1.03 (0.83-1.29)	0.513	1.09 (0.84-1.43)	0.823	1.02 (0.83-1.26)	0.569	1.04 (0.90-1.21)
Female												
Smoking[a]	235	43 (11.7)	128 (12.2)	64 (9.7)	0.119	0.79 (0.58-1.06)	0.791	0.95 (0.67-1.36)	0.211	1.19 (0.91-1.56)	0.229	0.89 (0.73-1.08)
Heavy smoking[a]	44	13 (3.5)	26 (2.5)	5 (0.8)	**0.006**	**0.27 (0.11-0.69)**	**0.041**	**0.50 (0.26-0.97)**	0.259	1.42 (0.77-2.60)	**0.002**	**0.50 (0.32-0.78)**
SI≤18[a]	155	29 (7.9)	85 (8.1)	41 (6.2)	0.146	0.76 (0.53-1.10)	0.726	0.93 (0.61-1.41)	0.276	1.20 (0.86-1.67)	0.239	0.87 (0.69-1.10)
Smoking[b]					0.109	0.78 (0.57-1.06)	0.747	0.94 (0.66-1.36)	0.212	1.20 (0.90-1.58)	0.212	0.88 (0.72-1.08)
Heavy smoking[b]					**0.006**	**0.27 (0.10-0.68)**	**0.037**	**0.49 (0.25-0.96)**	0.254	1.43 (0.77-2.63)	**0.002**	**0.49 (0.32-0.77)**
SI≤18[b]					0.141	0.76 (0.52-1.10)	0.694	0.92 (0.60-1.41)	0.284	1.20 (0.86-1.68)	0.225	0.86 (0.68-1.10)
Smoking[c]					0.117	0.78 (0.57-1.07)	0.736	0.94 (0.65-1.35)	0.229	1.19 (0.90-1.58)	0.218	0.88 (0.72-1.08)
Heavy smoking[c]					**0.006**	**0.27 (0.10-0.68)**	**0.035**	**0.49 (0.25-0.95)**	0.263	1.42 (0.77-2.62)	**0.002**	**0.49 (0.31-0.77)**
SI≤18[c]					0.140	0.76 (0.52-1.10)	0.681	0.91 (0.60-1.40)	0.288	1.20 (0.86-1.67)	0.219	0.86 (0.68-1.09)

a: Unadjusted

b:Adjusted for demographic characteristics (age, gender, education, occupation and ethnic)

c: Adjusted for demographic characteristics (age, gender, education, occupation and ethnic) , BMI and sports time

SI=age of smoking initiation

Supplementary Figure

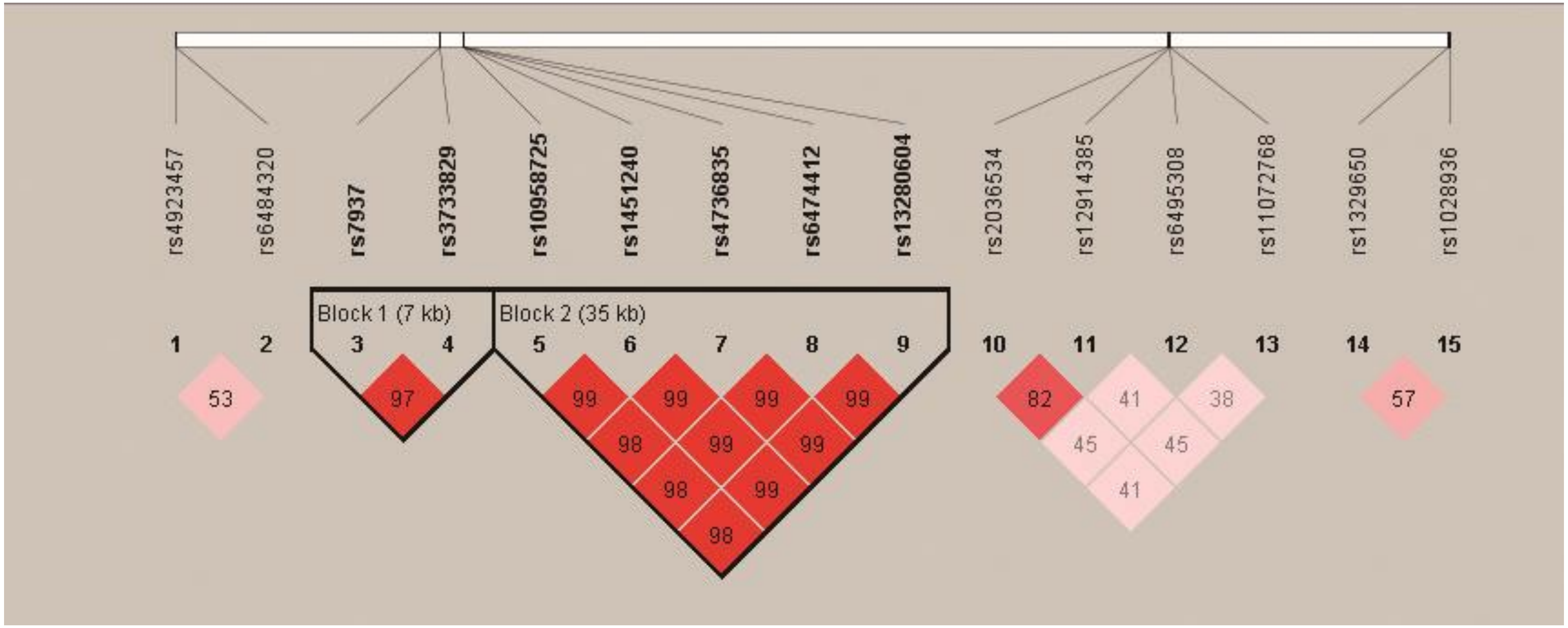

Supplementary Fig. S1. The LD plot of the 15 SNPs
